# Improving Whole Tomato Transformation for Prostate Health: Benign Prostate Hypertrophy as an Exploratory Model

**DOI:** 10.3390/ijms24065795

**Published:** 2023-03-17

**Authors:** Pier Giorgio Natali, Mauro Piantelli, Marco Minacori, Margherita Eufemi, Luisa Imberti

**Affiliations:** 1 Department of Medicine and Aging Sciences, Center for Advanced Studies and Technology (CAST), G. D’Annunzio University, 66100 Chieti, Italy; 2 Department of Biochemical Science “A. Rossi Fanelli”, Faculty of Pharmacy and Medicine, “La Sapienza” University of Rome, P. le Aldo Moro 5, 00185 Rome, Italy; 3 Section of Microbiology, University of Brescia, P. le Spedali Civili, 1, 25123 Brescia, Italy

**Keywords:** benign prostate hypertrophy, food supplement, prostate cancer, tomato

## Abstract

It is well-established that the beneficial properties of single phytonutrients can be better attained when they are taken with the complex of the molecules present in their natural milieu. Tomato, the fruit providing the most comprehensive complex of prostate-health-preserving micronutrients, has been shown to be superior to its single-nutrient counterparts in decreasing the incidence of age-related prostate diseases. Herein, we describe a novel tomato food supplement enriched with olive polyphenols, containing cis-lycopene concentrations far exceeding those present in industry-produced tomato commodities. The supplement, endowed with antioxidant activity comparable to that of N-acetylcysteine, significantly reduced, in experimental animals, the blood levels of prostate-cancer-promoting cytokines. In prospective, randomized, double-blinded, placebo-controlled studies performed on patients affected by benign prostatic hyperplasia, its uptake significantly improved urinary symptoms and quality of life. Therefore, this supplement can complement and, in some cases, be an alternative to current benign prostatic hyperplasia management. Furthermore, the product suppressed carcinogenesis in the TRAMP mouse model of human prostate cancer and interfered with prostate cancer molecular signaling. Thus, it may offer a step forward in exploring the potential of tomato consumption to delay or prevent the onset of age-related prostate diseases in high-risk individuals.

## 1. Introduction

The presence of chronic inflammation in an aging prostate may lead to two main diseases, benign prostatic hyperplasia (BPH) and cancer (PCa) [1]. BPH may severely impair the quality of life in one third of subjects older than 50 years and is reported in about 90% of individuals reaching 80 years of age [2]. PCa is the most frequently diagnosed cancer in men, with an annual incidence of nearly 1.5 million new cases [3]. Although significant differences in PCa incidence and mortality across ethnic groups have been reported [4], this malignancy represents an alarming public health issue worldwide because of the increasing incidence in young patients and the growing frequency of advanced disease cases [5]. In view of the global augmented life expectancy, both BPH and PCa embody a growing economic burden for wealthy societies and for patients’ direct and indirect costs [6,7].

Because of their late age occurrence, BPH and PCa are suitable for preventive interventions since even a modest delay in their development can lead to a substantial reduction in the incidence of clinically identifiable lesions. Primary prevention of BPH and PCa relies on healthy choices, such as regular exercise, low fat intake, and high vegetable and fruit consumption [8]. The latter recommendations, often inclusively described as adherence to the Mediterranean diet [9], advocate the regular uptake of various antioxidants and anti-inflammatory complexes present in fresh vegetables and fruits [10,11]. In this regard, tomatoes and olives are of great nutritional importance as they represent the main source of such complexes [12,13]. Accordingly, their consumption is associated with a low overall mortality rate [14,15].

## 2. Tomato and Olive Oil in Prostate Health

### 2.1. BPH’s Natural History and Current Medical Treatment

The molecular mechanisms underlying the pathogenesis of BPH leading to a symptomatic disease are still ill-defined [16]. Age-related endocrine [17] and vascular changes [18] as well as uncontrolled oxidative stress derived from acute and chronic inflammation have been proposed to induce the overgrowth of the stromal and cellular compartments in the transition zone of the gland [19,20,21]. However, because a low degree of proliferation of both epithelial and stromal cells has been detected, BPH has been interpreted as the result of impaired programmed cell death mechanisms [22,23]. Of interest, the recent molecular profiling of BPH has identified distinct subtypes, such as those with altered mTOR signaling linked to unfavorable prognosis [24], which are candidates for more targeted therapies.

The diagnosis of BPH is usually driven by lower urinary tract obstructive symptoms (LUTSs), i.e., urinary hesitancy, urgency, frequency, and post-void dribble [25].

Patients with BPH show a higher incidence of “in situ” PCa [26,27], thus suggesting that a reduction in risk factors or BPH treatment can, in at least a fraction of patients, delay the onset of PCa.

Chronic inflammation has also been shown to contribute to an increased risk of rapid disease progression because of a lower response to medical treatment [28]. Therefore, inflammation represents a key target of BPH treatment [29]. However, innovation in drug development for BPH is currently stalled, being confined to alpha-blockers, 5-alpha reductase inhibitors, and phosphodiesterase type 5 inhibitors [30,31] that, because of the underlying heterogeneity of BPH lesions [24,32], are often used in combination or successively [33].

Despite these strategies, LUTSs are not controlled in about one third of patients [34], and a number of responders’ side effects, such as decreased libido, erectile dysfunction, dizziness, and hypotension, may occur [33]. The failure of medical treatment invariably leads to surgical intervention to reduce LUTSs’ severity [35].

### 2.2. Phytotherapies for BPH: The Role of Tomato and Olive Micronutrients

Guidelines on therapeutic options for BPH include phytotherapy as well. Indeed, phytocompounds, either as plant portions, derived extracts, or purified molecules, are increasingly used in the treatment of patients with moderate–severe BPH [36]. A large body of epidemiological, experimental, and clinical studies on BPH prevention or improvement of the associated symptoms [37,38] has focused on tomato lycopene, the red-colored, open-chain beta carotenoid devoid of retinoid activity, present in a variable concentration in different tomato-cultivar-derived foods. Lycopene, which is contained in all tomato dietary sources, mainly in the low bioavailable trans isomeric form [39], is endowed with a large range of biological activities, which are also retained by its metabolites [40]. Its bioavailability, on the other hand, can be affected by a number of factors [39,41,42] and, differently from the trans isomeric form [43], the biologically active cis lycopene, which is produced mainly by heating the fruit [44], concentrates on definite anatomical sites, which include the prostate [39].

However, comparative studies have clearly demonstrated that the healthy properties of tomatoes could not be not exclusively ascribed to their lycopene content [45,46]. Indeed, a number of compounds endowed with wide-ranging biological activity are present in the fruit or are newly formed during its heat processing [12,47,48]; thus, the overall complex contributes to the in vivo anti-proliferative, anti-inflammatory, and pro-apoptotic activities [47,49,50], as well as to the anti-androgenic properties of the fruit [51]. As a result, the consumption of whole tomatoes has been shown to provide healthier effects than lycopene alone in experimental [10,45] and clinical studies [46,52].

Olive oil is well proven to represent a source of chemo-preventive nutrients [13]. Its polyphenol content prevents PCa development and invasiveness [53], as these molecules can modulate the molecular signaling involved in the growth and proliferation of transformed cells in inflammation (e.g., MAPK, PI3K, and NF-κB) [54] and angiogenesis [55]. In particular, hydroxytyrosol has been reported to inhibit in vitro human PCa cell proliferation and induce apoptosis [56,57].

### 2.3. Tomato and Olive Eco-Sustainability

Various phytochemicals endowed with prostate-healthy properties are widely present in nature [58]. We have concentrated our investigation on complexes of tomato and olive micronutrients, with our choice herein detailed.

Tomato, which represents the second most popular crop worldwide [59], has a high global consumption rate [60]. The fruit, characterized by extensive biodiversity [61] and chemodiversity [62], with a high average nutritional yield [63], requires timely controlled irrigation and moderate soil tillage [64]. Tomatoes accumulate low levels of environmental contaminants [65] in the fruit and are thus considered to be an “excluder plant” since they concentrate soil contaminants in the roots, stems, and shoots [64]. Residues of pesticides, if present on the skin, are removed by washing and cooking [66]. In addition, tomato lycopene has been shown to decrease the risk associated with exposure to natural chemical toxins, including pesticides and herbicides [67].

The waste caused by tomatoes’ industrial processing, as well as their packaging, is highly recyclable [68]. Because of their unique culinary versatility in multiethnic cuisine, they have wide acceptance in various dietary regimens.

Olive cultivation is another important agro-industrial sector [69] that also plays a role in maintaining biodiversity. Olive growth is possible under extreme environmental conditions, such as drought and high temperatures [70,71]. Epidemiological and experimental data have underscored the healthy properties of moderate dietary consumption of olive oil due to its antioxidant and inflammatory components [13,72].

### 2.4. Development of a New Whole-Tomato-Based Food Supplement (WTFS)

Among the different preparations derived from roots, seeds, pollen, bark, or fruits [73,74], the tomato is particularly attractive for prostate health maintenance. The fruit’s anti-inflammatory and antioxidant molecules [12], by acting both systemically as well as in the gland, are potentially useful in also shielding prostate cells [75] from aging-related degenerative changes [58].

There is no scientific evidence of any interaction between tomato and drugs, a relevant aspect in older patients who are often undergoing multiple pharmacologic treatments.

Because the bioavailability of lycopene, the tomato’s major antioxidant, is highly increased by cooking the fruit [42] and by the presence of fats, especially olive virgin oil [76], early attempts to improve tomato antioxidant activity used a concentrated whole ripe fruit puree heated at 95 °C for 5 min, with 10% extra-virgin olive oil added. This “food for special medical purposes” (FSMP), when administered to patients affected by chronic viral C hepatitis, significantly increased plasma lycopene concentrations compared to other tomato products and was thus effective in preventing carotenoid serum depletion and in improving the oxidative status during antiviral therapy [77].

When tested in a transgenic mouse model of human prostate carcinogenesis (TRAMP) [78], the supplementation of the animal’s diet with 10% of FSMP significantly decreased the appearance of poorly differentiated cancer and mortality [79]. In addition, an in-depth serological analysis of the animals during treatment demonstrated that the FSMP was able to reduce the levels of circulating inflammatory/angiogenic cytokines, such as vascular endothelial growth factor (VEGF), tumor necrosis factor alpha (TNF-α), and interleukin (IL)-6 [79]. These effects were dose-dependent since tomato-based supplemented diets with lower lycopene concentrations failed to modify the clinical course of the TRAMP mice’s cancer [80].

**Figure 1:**
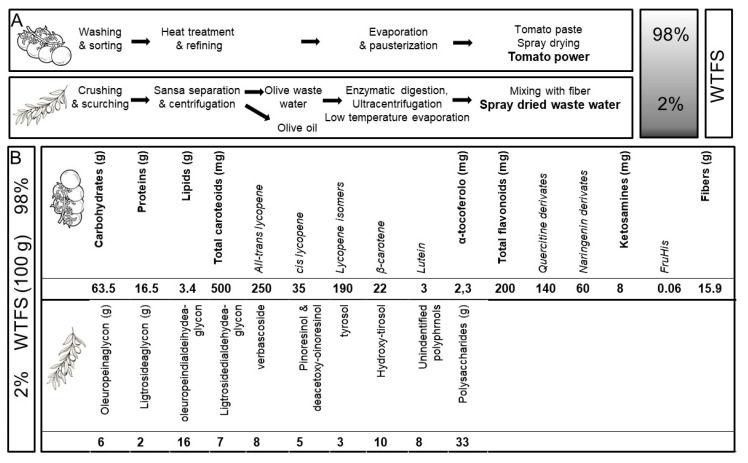
(**A**) Protocol for “whole tomato food supplement” (WTFS) preparation. (**B**) Composition for 100 g of WTFS.

Along this line of investigation, and with the aim of producing an improved and standardized “whole tomato food supplement” (WTFS) of potential use in clinical studies, an innovative whole tomato, solvent-free processing protocol [81] described in Figure 1A has been developed.

When FSMP and WTFS compositions were compared, the new production method resulted not only in higher concentrations of tomato antioxidant and anti-inflammatory micronutrients, such as carotenoids (lycopene, beta-carotene, and lutein), but also in the formation of d-fructose- l-histidine (Fru-His) and ketosamine, which increased the biological activity of the carotenoid [48] (Figure 1B). Of relevance, a large fraction of lycopene is now present in the more bioavailable and biologically active cis configuration [39].

**Figure 2:**
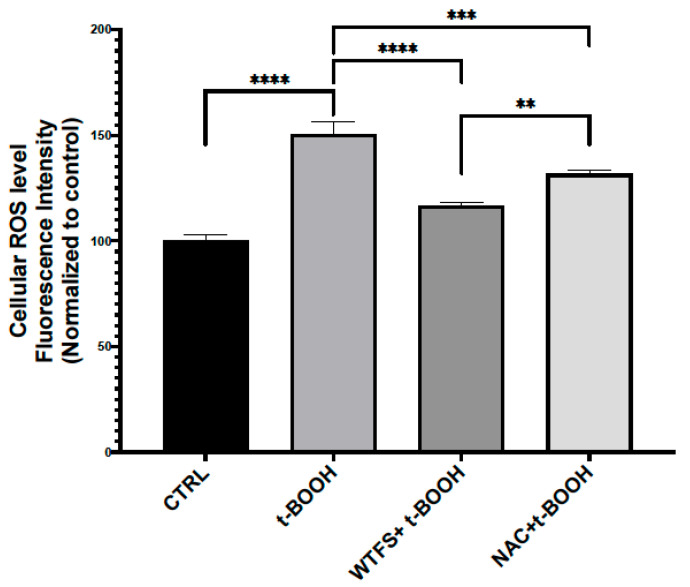
Reactive oxygen species (ROS) detection. To compare the antioxidant activity of WTFS and N-acetylcysteine (NAC), LNCaP (androgen-sensitive human prostate epithelial cell line) cells were pretreated for 4 h with 1 mg/mL of WTFS or 500 µM of NAC and then were stressed with 75 μM of tert-butyl hydroperoxide (t-BOOH) for 30 min. ROS were quantified using the CellROX Green Flow Cytometry Assay Kit (Thermo Fisher Scientific, Monza, Italy, cat. C10492) following the manufacturer’s instructions. Samples were analyzed using a BD Accuri C6 flow cytometer (BD Biosciences, Milano, Italy). The experiment was repeated three times with similar results, and the obtained values are presented as the mean and standard deviation. Statistical analysis was performed using GraphPad Prisma 8.2.1(279) software and ANOVA followed by Tukey’s post hoc test. Statistically significant differences (** *p* < 0.01; *** *p* < 0.001; **** *p* < 0.0001) are marked with asterisks and refer to the untreated LNCaP cells used as the control (CTRL).

With the dual aim of protecting the carotenoid content from oxidative degradation and increasing the WTSF of prostate-health-relevant molecules with minor caloric uptake [55,82], 2% of olive wastewater (OWW) was added. Thus, both tomato (i.e., naringenin and quercetin) and OWW (i.e., oleuropein, tyrosol, hydroxytyrosol, pinoresinol, and verbascoside) polyphenols contribute to WTFS composition. As a result, the WTFS displayed antioxidant activity comparable to that of N-acetylcysteine (as shown in the unpublished data reported in Figure 2), one of the most frequently used antioxidant drugs, and was more active than the FSMP in reducing the serum levels of interleukin (IL)-6 and VEGF in the TRAMP mouse model [81].

To our knowledge, WTFS is the only available food supplement with lycopene concentrations exceeding those present in tomato-based consumer products [83] and with known concentrations of cis lycopene and other micronutrients of the fruit.

### 2.5. WTFS in BPH

The availability of the standardized WTFS product allowed for its assessment in a clinical pilot study which demonstrated that following the daily assumption of 6 gr of the WTFS containing 22 mg of total carotenoids for two months, 80% of symptomatic BPH patients showed a decrease in LUTSs [84]. The patients’ compliance was high, and no side effects were reported. Therefore, a phase II prospective, randomized, double-blinded, placebo-controlled study was performed on patients with biopsy-proven BPH characterized by various degrees of inflammation [85]. The results of this trial indicated that following the same treatment schedule as the pilot study, WTFS significantly relieved LUTSs (*p* < 0.0002) and improved the quality of life (*p* < 0.0001). In this context, the WTFS uptake did not change free-prostate-specific antigen (PSA) and free/total PSA ratio values, but a trend in the decrease in free PSA in patients with baseline levels above 10 ng/mL was documented [86,87]. This is in line with data from early studies demonstrating that tomato supplementation reduces PSA levels, but just in PCa patients [88].

Only one patient left the study, and no side effects, often associated with the culinary use of tomato [89], were recorded.

In view of the fact that metabolic syndrome and chronic inflammation, both considered to be risk factors for BHP, are frequently observed in HIV-infected patients [90] (in whom PCa incidence is expected to increase in the near future [91]), a validation study was performed on HIV+ patients with BPH using the treatment schedule. Additionally, in this trial, the daily WTFS consumption resulted in a significant amelioration in LUTSs, quality of life, free/total PSA ratio, and diastolic blood pressure, with a trend in the decrease in the IL-6 serum level [92].

### 2.6. Links between BPH and Pca

BPH and PCa share genetic traits [93]. Epidemiologic and pathologic links between BPH and later PCa development have been reported, especially for Asian patients [94,95], that parallel those described by a European long-term, large cohort study [96]. As in BPH, PCa may be associated with metabolic syndrome and insulin resistance, suggesting a relationship with a dietary factor for this group of diseases [97]. This is also supported by the association between high body mass index, an element of metabolic syndrome, and PCa progression and specific mortality [98,99,100,101,102].

**Figure 3:**
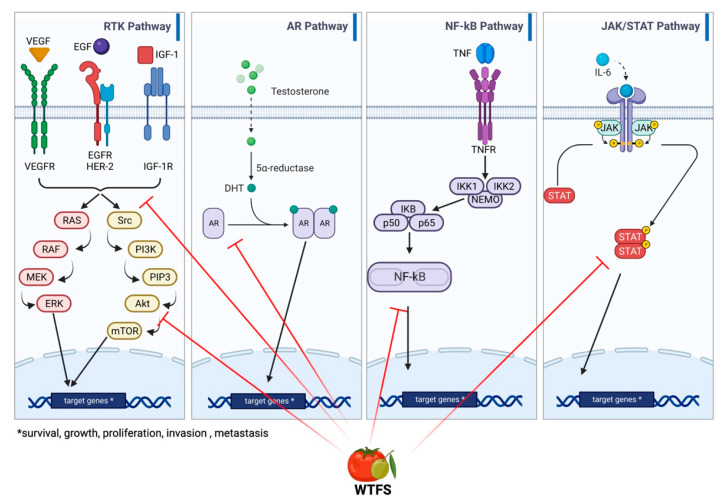
Main signaling pathways involved in BHP and PCa development: receptor tyrosine kinase (RKT), androgen receptor (AR), NF-κB, and JAK/STAT signaling. WTFS targets (also see Table 1) are highlighted by red lines.

Although the understanding of the molecular basis of the two diseases is far from being fully outlined, common deranged molecular pathways include hormonal dependence [103], chronic inflammation, and downstream signaling pathways involving cytokines such as IL-12, TNF-α, IL-1, IL-1β, and IL-6 [104,105]; downstream signaling pathways mediated by NF-κB, which contributes to tumor progression [106]; IGF1R-modulating angiogenesis [107]; and IL-6/JAK/STAT signaling, which stimulates cell growth and impairs apoptosis [108,109]. An overview and detailed representation of the common pathways between BPH and PCa are represented in Figure 3.

Collectively, the above information supports the hypothesis that the two diseases offer a number of molecular targets druggable in adjuvant settings by complexes of phytonutrients [110], such as those present in tomatoes and olives.

### 2.7. Tomato Consumption and PCa

Due to the above relationships, phytotherapies are also gaining increasing attention in PCa management in preventive, therapeutic, and palliative settings [58]. A link between tomato and lycopene uptake and PCa risk was initially indicated by epidemiologic studies [88,111,112]. A prospective study of tomato products confirmed the inverse association between tomato sauce consumption and PCa risk. Notably, subjects consuming more than two servings/week of tomato compared to less than one serving/month exhibited a 66% decreased risk of PCa metastatic cancer [113]. More recently, a consistently high intake of tomato after PCa diagnosis was found to be associated with a significantly lower risk of specific mortality among patients diagnosed with high-risk tumors [114]. In addition, low lycopene concentrations in the prostate favor PCa onset in patients with high-risk prostatic intraepithelial tumors [115]. Conclusively, a meta-analysis confirmed that the beneficial effect of processed (cooked tomatoes and sauces) and raw tomato consumption on PCa risk are dose-dependent, and adherence to the PCa-specific dietary recommendations via constant tomato consumption is associated with a decreased risk of PCa [89].

### 2.8. WTBS and Inhibition of PCa-Activated Molecular Pathways

Early evidence that WTBS can interfere with prostate carcinogenesis is derived from the TRAMP murine carcinoma model of progressive PCa that mirrors the stages of human disease, including the androgen-independent stage [79]. In these mice, the FSMP diet significantly increased overall survival (*p* < 0.01), delayed progression from prostatic intraepithelial neoplasia to adenocarcinoma, and decreased the incidence of poorly differentiated cancer. Biochemical studies disclosed a decrease in antioxidant activity in animal sera and a reduction in the circulating biomarkers of relevance to prostate carcinogenesis, such as IL-6 and TNF-α, which significantly correlated with PCa grade [116] and VEGF, known to be associated with prostate tumor grade, metastasis, and prognosis [117]. Indeed, the IL-6/STAT axis represents a link between inflammation and prostate carcinogenesis [105], which is the signal transducer and activator of transcription 3 (STAT3), a key modulator in the expression of a wide range of oncogenic genes [118] and a player in prostate cancer energy [119].

Furthermore, recent data created using the androgen-sensitive human prostate epithelial cell line LNCaP exposed to an environmental carcinogen have demonstrated that WTFS protects DNA from oxidative stress damage, blocks the pathways involved in PCa development—such as STAT3 activation androgen receptor signaling—and displays pro-apoptotic and anti-proliferative properties [120]. Table 1 summarizes the biological activities of the complex of micronutrients present in the WTFS.

**Table 1:** Activity of WTBS’s single components.

Compound	Activity	References
**Lycopene**		[87,88,121,122,123]
	- Antioxidant	
	- Anti-proliferative activity	
	- Apoptosis induction	
	- Protection against DNA damage	
	- Androgen receptor transcriptional inhibition	
	- Decreased PSA levels	
	- STAT3 inhibition	
**Tyrosol/hydroxytirosol**		[57,124,125,126]
	- Antioxidant	
	- Protection against DNA damage	
	- Reduced androgen receptor expression	
	- Decreased PSA levels	
	- STAT3 inhibition	
**Tocopherol**		[127,128,129,130]
	- Apoptosis induction	
	- AR transcriptional inhibition	
	- Decreased PSA levels	
	- STAT3 inhibition	
**Quercetin**		[131,132,133]
	- Antioxidant	
	- Anti-proliferative activity	
	- Apoptosis induction	
	- Protection against DNA damage	
	- STAT3 inhibition	
**Secoiridoid aglycones (oleuropein, ligstroside)**		[134,135,136,137]
	- Anti-proliferative activity	
	- Apoptosis induction	
**Verbascoside**		[138,139,140]
	- Apoptosis induction	
	- Protection against DNA damage	
	- STAT3 inhibition	
**Pinoresinol**		[141,142]
	- Antioxidant	
	- Anti-proliferative activity	
	- Protection against DNA damage	

The presence of intact plant miRNAs in human tissues has been clearly demonstrated, suggesting their dietary origin and thus raising the question as to whether they can modulate human gene expression [143]. In this regard, the WTFS has been shown to contain miR171 (unpublished data by Andrea Galgani, Tor Vergata University of Rome, Italy) capable of modulating the mTOR pathway involved in PCa development [144,145].

## 3. Discussion and Future Directions

Although numerous plant compounds have revealed bioactive properties, including antioxidant, anti-inflammatory, and anticancer, the combination of the tomato and the olive has several advantages: (a) none of the other “plant compounds” have the properties of tomato and olive in terms of fulfilling the criteria that we have referred to in the section “Tomato and olive eco sustainability” [13,58,59,60,61,62,63,64,65,66,67,68,69,70,71,72,73,74], representing a limitation in their use as functional foods; (b) a dose-dependent effect has been demonstrated only for a limited number of plants; (c) none of the other plant compounds are known to concentrate in the prostate and to provide the complex of molecules with converging biological effects such as those summarized in Table 1; and (d) when dealing with aging-related prostate diseases, the WTBS is also endowed with anti-androgen receptor inhibition [120].

Experimental and clinical evidence have demonstrated that the newly developed tomato processing method results in a WTFS, which is enriched in more bioavailable lycopene cis isoforms and in new nutrients, i.e., ketosamines, able to increase the biological activity of the carotenoid. Currently, the WTFS is the only available supplement for humans containing the highest concentrations of lycopene, especially in the cis isoform, compared to available tomato-based consumer products.

The addition of olive polyphenols results in a food supplement endowed with high antioxidant activity comparable to that of NAC and, in animal models, opposes the production of inflammation-associated cytokine. In phase II, prospective, randomized, double-blinded, placebo-controlled studies performed on patients affected by benign prostatic hyperplasia, the uptake of the WTFS resulted in a significant improvement in LUTSs and quality of life with no side effects.

Ongoing investigations are now addressing clinically relevant open questions regarding the duration of the efficacy of WTFS in controlling LUTSs, the long-term effects of the treatment in the natural story of BPH, and the exploratory trial of WTBS associated with other current BPH therapies.

Furthermore, the supplement may be a candidate of choice for exploring its preventive and therapeutic activities in prospective clinical trials, especially in high-risk BPH patients such as those affected by metabolic syndrome and those with altered mTOR signaling. In this regard, the WTFS, with a low caloric content (334 kcl/100 mg), may represent an ideal food supplement to complement culinary tomato consumption.

The WTBS can block the natural history of PCa in animal models mimicking human diseases as well as molecular pathways supporting malignant prostate transformation.

The considerable cost and complexity of cancer prevention prospective clinical trials using complexes of phytocompounds rather than single nutrients may be facilitated by the availability of standardized supplements. On the basis of the available knowledge, the present WTFS may offer a new tool to explore, without confounding factors, the actual potential and limits of tomatoes to prevent or delay the onset of PCa in high-risk individuals.

Although it is in the preliminary stages of investigation, the WTBS may represent a candidate to explore population-focused remediation strategies focused on environmental carcinogenic contaminants.

## Data Availability

Data sharing is not applicable to this article.
